# Different risk factors distinguish myalgic encephalomyelitis/chronic fatigue syndrome from severe fatigue

**DOI:** 10.1038/s41598-023-29329-x

**Published:** 2023-02-11

**Authors:** Natalia Palacios, Samantha Molsberry, Kathryn C. Fitzgerald, Anthony L. Komaroff

**Affiliations:** 1 Department of Public Health, Zuckerberg College of Health Sciences, University of Massachusetts Lowell, 61 Wilder Street, O’Leary Library, Suite 540-K, Lowell, MA 01854 USA; 2 Geriatric Research Education Clinical Center, Edith Nourse Rogers Memorial Veterans Hospital, Bedford, MA USA; 3 Department of Nutrition, Harvard School of Public Health, Boston, MA USA; 4 Department of Epidemiology, Harvard School of Public Health, Boston, MA USA; 5 Department of Neurology, School of Medicine, Johns Hopkins Bloomberg School of Public Health, Baltimore, MD USA; 6 Division of General Medicine, Department of Medicine, Brigham and Women’s Hospital, Harvard Medical School, Boston, MA USA; 7 Center for Solutions for ME/CFS, Columbia University, New York, USA

**Keywords:** Medical research, Risk factors

## Abstract

Fatigue is a common reason that patients seek medical care. Only a fraction of these patients meet criteria for myalgic encephalomyelitis/chronic fatigue syndrome (ME/CFS). To determine if ME/CFS is just a more extreme form of fatigue, or a qualitatively different condition, we assessed whether risk factors for ME/CFS and for Severe Fatigue were similar. An email questionnaire that inquired about symptoms of Severe Fatigue and ME/CFS was completed by 41,802 US female nurses from whom detailed medical and lifestyle information had been collected since 1989: 102 met criteria for ME/CFS, 522 had Severe Fatigue, and 41,178 individuals were without significant chronic fatigue. We used Cox proportional hazards regression to estimate the Hazard Ratio (HR) of Severe Fatigue and of ME/CFS with each of several potential risk factors, according to the level of exposure to each risk factor. The risk of Severe Fatigue was significantly increased among participants who were older, had a higher BMI in adulthood, used hormone therapy, had increased alcohol intake and decreased caffeine intake. In contrast, these risk factor associations were not seen in people with ME/CFS. A self-reported past history of acute infectious mononucleosis was associated with a non-significantly increased Hazard Ratio of later ME/CFS (HR 1.77, 0.87–3.61) and, to a lesser extent, of Severe Fatigue (HR 1.28, 0.98–1.66). The different contribution of various risk factors to Severe Fatigue and ME/CFS suggests that ME/CFS has a qualitatively different underlying biology from the more common state of Severe Fatigue.

## Introduction

Myalgic encephalomyelitis/chronic fatigue syndrome, or ME/CFS (the illness formerly called chronic fatigue syndrome) is a debilitating illness that severely impacts the quality of life. The Institute of Medicine/National Academy of Sciences and the Centers for Disease Control and Prevention (CDC) estimate that between 836,000 and 2.5 million people in the United States have ME/CFS and that the illness leads to direct and indirect economic costs of between $17–24 billion annually in the U.S.^1,2^. Research over the past 35 years has identified underlying biological abnormalities involving the central nervous system, autonomic nervous system, and energy metabolism, as summarized recently at a conference convened by the National Institutes of Health^3^.

The potential risk factors that have been investigated with relation to ME/CFS include history of infections^4^, particularly acute infectious mononucleosis (hereafter, simply “mononucleosis”)^5–12^, and lifestyle factors including body mass index (BMI)^13^, physical activity^14^ and sedentary behavior^15^. Smoking does not appear to be a risk factor^15,16^. Results of studies examining these risk factors have been inconsistent, in part because many studies had relatively small sample sizes. In addition, these studies typically followed patients for no longer than two years after the onset of mononucleosis. In this study, in contrast, we followed a large prospective cohort of over 40,000 female nurses for 20 years.

## Methods

### Study population and study design

The Nurses’ Health Study II (NHS II) is a prospective cohort of 116,700 female registered nurses, 25–45 at baseline, enrolled in 1989 and followed biennially ever since^17,18^. Obviously, the cohort is not representative of the population at large. However, since the cohort consists of health professionals, the participants can be expected to accurately report health information. Lessons learned from the Nurses’ Health Study cohort have been the subject of several thousand publications.

### Window of observation

The NHS II cohort was enrolled in 1989 (referred to as “baseline” in this report), and the last full biennial questionnaire available to this study was in 2009. Thus, the follow-up spanned 20 years. In addition to reporting risk factors at baseline and biennially thereafter, study participants also reported their memory of some risk factors when they were age 18. The dates of onset of ME/CFS and Severe Fatigue were ascertained through 2009. The protocols were approved by the Institutional Review Board (IRB) of Brigham and Women’s Hospital.

### Survey instruments

#### Full questionnaire

Every 2 years, all NHS II participants complete an extensive *full questionnaire* that includes detailed information about lifestyle factors, diet, medication use and diseases and medical conditions, as previously described^19^.

#### Online questionnaire

In addition, in 2009, a fatigue-specific online questionnaire was sent to all NHS II participants who had provided email addresses including: (1) questions relevant to the CDC criteria for ME/CFS or for Severe Fatigue, including the date of onset of fatigue and whether the participant had ever (and when) received a diagnosis of ME/CFS from a physician; (2) risk factors—as described in more detail below.

### Ascertainment criteria for the ME/CFS group

Data from the two survey instruments were used to identify cases of ME/CFS according to the 1994 CDC criteria^20^.

#### Definition of severe fatigue

The CDC criteria require, first, that a patient have at least six months of severe fatigue. Participants were asked five questions: (1) Are you currently suffering from fatigue (lack of energy or tiredness) severe enough that it has caused you to substantially reduce your regular activities, at work or at home? (2) Has your fatigue persisted (constantly or on and off) for > 6 months? (3) Is this fatigue a change from the way you felt previously? (4) Did your fatigue have a definite onset? (5) Is your fatigue substantially alleviated by rest? Participants who answered “Yes” to the first four questions and “No” to the last were judged to meet the first major criterion for ME/CFS. If they did not also have the “additional ME/CFS symptoms” (listed just below) they were classified as having Severe Fatigue.

#### Additional ME/CFS symptoms

If a patient met the first major criterion for ME/CFS, and also had at least four of the following eight chronic symptoms for at least six months—impaired memory and concentration, sore throat, unrefreshing sleep, multi-joint pain, neck or axillary adenopathy, muscle pain, new headaches, and post-exertional malaise—they were considered strong candidates for having ME/CFS. The online questionnaire explicitly asked about each of these symptoms.

#### Determination of exclusionary diagnoses

Finally, the CDC criteria for ME/CFS require that a person also must have no other exclusionary illness that could explain the fatigue. The NHS II full questionnaire, completed every two years, explicitly asked about diseases that, if active and uncontrolled near the onset of the fatigue, would constitute exclusionary conditions: systemic lupus erythematosus, multiple sclerosis, thyroiditis, myasthenia graves, depression, angina, myocardial infarction, ulcerative colitis, tuberculosis, asthma, coronary artery bypass graft. The full questionnaire did not distinguish melancholic depression or unipolar psychotic depression from other forms of depression.

When a potentially exclusionary diagnosis was reported, we compared the date of onset (the two-year window since the last full questionnaire was administered) to the date of onset of the fatigue (as reported on the online questionnaire). When an exclusionary diagnosis had developed in the window of time beginning two years before and ending two years after the self-reported date of fatigue onset, the participant was considered *not* to meet the criteria for ME/CFS. An exception to this rule was depression: people were excluded from the study only if their depression had been diagnosed *before* the onset of fatigue. Because we were unable to determine if such preexisting depression was categorized as melancholic or unipolar psychotic depression (the types of depression that were clearly exclusionary), we conservatively treated all types of preexisting depression as exclusionary.

The online questionnaire asked about several exclusionary diagnoses that were not included in the full questionnaire—chronic hepatitis, narcolepsy, sleep apnea, bulimia and anorexia nervosa. Because we asked this question only once (not every two years), and because these conditions were not easily controlled by medications, we regarded them as exclusionary diagnoses whenever they were reported.

### Ascertainment criteria for the severe fatigue and not fatigued groups

Participants not classified as being in the ME/CFS group could be classified either in the Severe Fatigue or the Not Fatigued groups. Membership in the Severe Fatigue group required meeting the same severity of fatigue as the ME/CFS group, but *not* the additional ME/CFS symptoms, and to have had the onset of their fatigue begin between 1989 and 2009.

All subjects who were neither in the ME/CFS group nor the Severe Fatigue group were included in the Not Fatigued group; thus, the three groups were mutually exclusive and collectively exhaustive.

### Ascertainment of exposures (risk factors) and other covariates

The variables to be analyzed were selected a priori, based on a review of the literature and the available data items in the cohort. Age (birthday), current height, current weight, weight at age 18, current physical activity, physical activity at ages 18–29, smoking status and quantity (pack years) and time spent sitting were assessed at baseline in 1989, when all respondents had reached adulthood. Caffeine and alcohol intake, in grams, was assessed in 1991, on the first dietary questionnaire in this cohort. The use of hormone therapy and menopausal status were also assessed biennially and updated in the analyses. BMI in 1989 was calculated as weight in kilograms in 1989 divided by height in meters squared; BMI at age 18 was calculated based on self-reported weight at age 18, assuming that height does not change significantly after age 18. Physical activity was assessed at ages 18–22 by questions about the frequency of strenuous activity, and it was assessed in adulthood both by estimating metabolic equivalents (METs), and by asking about hours per week sitting at home.

In 2001 a question of “Have you ever had infectious mononucleosis?” was asked of all cohort participants, with the answer options of ‘yes’, ‘no’ and ‘not sure’. The participants were then asked about the approximate age at which they had mononucleosis. However, fewer than half the women who responded to this question provided the age at mononucleosis. Those who did not were excluded from our analyses.

### Statistical analyses

To identify whether risk factors for ME/CFS differ from those for Severe Fatigue, we conducted separate parallel analyses for each of these outcomes. We used Cox proportional hazards models to estimate the Hazard Ratio and 95% confidence intervals (95% CI) for both ME/CFS and Severe Fatigue, according to the level of each risk factor.

We examined the assumption of proportional hazards for our models by adding a predictor by time interaction term to each of our models and examining their significance using the Likelihood Ratio Test. None of the interactions were significant (p < 0.05), leading us to conclude that the proportional hazards assumption held for all predictors in our analyses.

The outcome in the Cox model was the time to onset of ME/CFS or Severe Fatigue, as reported on the 2009 online questionnaire. Person time of follow-up for each participant in the survival analyses was computed from baseline (1989) until the date of onset of ME/CFS or Severe Fatigue (as reported on the online questionnaire), or until the end of follow-up in 2009, whichever came earlier. Statistical analyses were performed using SAS 9.1 (Cary, NC).

BMI in 1989, BMI at age 18 and physical activity in 1989 were analyzed in quartiles. The other variables were analyzed as categorical variables with cutoffs based on the distribution of each variable in the cohort. Models of the use of hormone therapy and of menopausal status used the time of onset of menopause as reported on the survey just before the reported onset of fatigue, and were applied to determine if the fatigue was related to menopause.

The Hazard Ratios for ME/CFS and for Severe Fatigue compared those with a self-reported history of infectious mononucleosis to a referent group without that history. The comparisons were adjusted for age, physical activity (quartiles), BMI (< 18.5, 18.5–24.9, 25–29.9, ≥ 30), and smoking status (never, past, current).

### Ethics approval and consent to participate

The institutional review board at Brigham and Women’s Hospital approved the study, all research was performed in accordance with relevant guidelines/regulations and in accordance with the Declaration of Helsinki, and written informed consent was obtained from all participants and/or their legal guardians.

## Results

### Data collected

The online questionnaire was e-mailed to 61,379 women and a reminder e-mail was sent to non-respondents. After two rounds of emails, a total of 41,802 nurses completed the online questionnaire—a 68% response rate. As described previously^19^, the respondents were generally representative of the NHS II cohort with respect to age, BMI and smoking exposure, were primarily Caucasian, and were more likely to be post-menopausal.

### Comparison of the ME/CFS, severe fatigue and not fatigued groups

Table 1 shows the study baseline characteristics of the three groups: ME/CFS (N = 102), Severe Fatigue (N = 522) and Not Fatigued (N = 41,178).

**Table 1:** Characteristics of NHS II participants who completed the fatigue questionnaire.

	ME/CFS (n = 102)	Severe fatigue (n = 522)	Not fatigued (n = 41,178)
Age in 1989 (years)^a^	33.76 (4.56)	34.88 (4.30)	35.08 (4.61)
BMI at age 18 (kg/m^2^)	21.20 (1.70)	21.33 (3.28)	21.22 (3.21)
BMI in 1989 (kg/m^2^)	24.32 (3.01)	24.75 (5.02)	23.87 (4.84)
Physical activity in 1989 (METs/week)^b^	28.18 (23.86)	22.73 (27.46)	23.99 (34.32)
Participation in strenuous physical activity at ages 18–22			
Never, %	34.59	31.07	29.23
1–3 months/year, %	20.27	27.11	30.81
4–6 months/year, %	9.91	17.80	16.81
≥ 7 months/year, %	35.24	24.02	23.15
Hours/week spent sitting at home in 1989			
< 5 h, %	23.02	32.41	28.62
6–10 h, %	22.08	25.31	24.11
11–20 h, %	26.71	24.03	27.18
≥ 20 h, %	28.20	18.25	20.09
Current smoker in 1989, %	15.76	15.91	11.19
Packyears smoked in 1989 (years)	11.33 (4.20)	13.49 (8.94)	11.17 (8.02)
Currently married in 1989, %	73.06	73.18	78.30
Caucasian race%	94.08	97.67	97.08
Employed in 1989, %	66.69	61.98	82.14
Hours worked per week in 1989			
Less than 15 h, %	0.76	2.81	3.39
More than 60, %	2.18	1.46	3.06
Normal, %	97.06	95.74	93.55
History of night shifts in 1989			
Never, %	44.93	39.35	39.20
1–5 years, %	43.82	48.99	48.59
5–10 years, %	6.31	7.50	8.08
> 10 years, %	4.94	4.16	4.13
Alcohol intake g/day in 1991	2.89 (3.72)	2.29 (3.45)	3.27 (6.16)
Caffeine intake mg/day in 1991	233.22 (107.49)	220.02 (194.41)	234.07 (209.74)
Postmenopausal in 1989, %	2.85	5.01	1.99
Current use of hormone therapy in 1989, %	2.85	4.42	1.82
Duration of oral contraceptive use in 1989 (months)	59.89 (87.60)	74.22 (146.26)	61.22 (127.28)
History of mononucleosis in 2001			
No, %	67.29	71.81	78.90
Yes, %	23.29	24.53	17.93
Unsure, %	9.41	3.66	3.17

Values are means (SD) or medians (Q25, Q75) for continuous variables; percentages or ns or both for categorical variables and are standardized to the age distribution of the study population.

Values of polytomous variables may not sum to 100% due to rounding.

BMI, body mass index.

^a^Value is not age adjusted.

^b^Metabolic equivalents (METs) from recreational and leisure-time activities.

ME/CFS group subjects, compared to the Not Fatigued group subjects, were slightly younger, had similar levels of BMI both at age 18 and at study baseline, had lower levels of alcohol intake, and had higher levels of physical activity when young and at baseline (before they became ill). A lower proportion of the ME/CFS and Severe Fatigue groups reported being employed, compared to Non Fatigued subjects.

Compared to the Severe Fatigue group subjects, the ME/CFS group subjects were more physically active when young and at baseline, were less likely to be postmenopausal.

### Impact of various risk factors

#### Age

While the mean ages of the subjects in the three groups were quite similar (Table 1), there was a very different relationship of age to risk in the two fatigued groups (Table 2). Increasing age was strongly related to increased risk of Severe Fatigue (HR 4.37, 2.99–6.39, when comparing participants over 55 to those 40 years and younger). In contrast, the risk of ME/CFS did not increase with increasing age.

**Table 2:** Age in 1989 and risk of ME/CFS and Severe Fatigue.

	ME/CFS			Severe fatigue		
	Cases	Person-years	HR (95% CI)	Cases	Person-years	HR (95% CI)
Age in 1989 (years)						
< 40	35	251,067	1.00 (ref)	65	251,534	1.00 (ref)
40–44	31	206,866	1.07 (0.66–1.74)	113	206,784	2.11 (1.56–2.87)
45–49	20	181,384	0.79 (0.46–1.37)	172	180,960	3.68 (2.77–4.89)
50–54	13	116,875	0.80 (0.42–1.51)	127	116,247	4.23 (3.14–5.70)
≥ 55	3	40,041	0.54 (0.17–1.75)	45	39,831	4.37 (2.99–6.39)

HR, hazard ratio.

#### Body mass index

BMI in adulthood (1989) was strongly associated with Severe Fatigue (p-trend < 0.001) and marginally associated with ME/CFS (p-trend = 0.04). BMI at age 18 was associated neither with ME/CFS nor Severe Fatigue (Table 3).

**Table 3:** Hazard ratio by risk factor, comparing ME/CFS to severe fatigue.

Risk Factor	ME/CFS				Severe fatigue			
	Cases*	Person-years	Multivariate-adjusted HR (95% CI)^†^	P_trend_	Cases	Person-years	Multivariate-adjusted HR (95% CI)^†^	P_trend_
BMI, age 18 (kg/m^2^)^†^								
< 18.5	10	111,768	0.63 (0.32–1.21)	0.67	78	111,602	1.12 (0.88–1.43)	0.18
18.5–24.9	84	600,945	1.00 (ref)		375	600,610	1.00 (ref)	
25.0–29.9	4	59,853	0.51 (0.19–1.40)		48	59,683	1.24 (0.92–1.68)	
≥ 30	3	17,663	1.14 (0.36–3.66)		20	17,484	1.68 (1.06–2.65)	
BMI in 1989 (kg/m^2^)								
< 18.5	1	25,764	0.32 (0.04–2.28)	0.04	22	25,786	1.55 (1.00–2.40)	< 0.0001
18.5–24.9	67	541,955	1.00 (ref)		302	541,714	1.00 (ref)	
25.0–29.9	19	141,449	1.17 (0.70–1.95)		106	141,123	1.37 (1.10–1.71)	
≥ 30	15	82,941	1.66 (0.94–2.93)		88	82,565	1.91 (1.50–2.43)	
Physical activity in 1989 (METs/week)^†^								
< 5.4	22	198,850	1.00 (ref)	0.88	149	198,370	1.00 (ref)	0.68
5.4–13.6	34	198,448	1.52 (0.89–2.62)		117	198,151	0.80 (0.63–1.02)	
13.7–29.4	17	198,198	0.77 (0.41–1.45)		125	198,451	0.87 (0.69–1.11)	
≥ 29.5	29	198,464	1.33 (0.75–2.34)		128	198,120	0.92 (0.72–1.17)	
Participation in strenuous physical activity at ages 18–22								
Never, %	35	231,849	1.00 (ref)	0.98	156	231,502	1.00 (ref)	0.81
1–3 months/year	22	243,708	0.55 (0.32–0.94)		153	243,410	0.90 (0.72–1.13)	
4–6 months/year	12	133,338	0.51 (0.26–0.99)		87	132,976	0.96 (0.73–1.25)	
≥ 7 months/year	33	183,330	1.00 (0.61–1.64)		124	183,476	1.00 (0.79–1.28)	
Hours/week spent sitting at home in 1989								
< 5 h	22	224,470	1.00 (ref)	0.04	159	224,068	1.00 (ref)	0.06
6–10 h	20	188,667	1.07 (0.58–1.98)		133	188,674	0.97 (0.77–1.22)	
11–20 h	31	212,666	1.44 (0.83–2.50)		129	212,646	0.85 (0.67–1.07)	
≥ 20 h	27	157,605	1.68 (0.95–2.97)		96	157,192	0.81 (0.63–1.05)	
Smoking status in 1989								
Non-smoker	66	526,901	1.00 (ref)		308	526,407	1.00 (ref)	
Past	19	179,031	0.89 (0.53–1.49)	0.64***	134	178,864	1.27 (1.03–1.56)	0.02***
Current	16	89,571	1.48 (0.85–2.57)	0.16***	79	89,333	1.47 (1.15–1.89)	.002***

	ME/CFS				Severe fatigue			
	Cases*	Person-years	Multivariate-adjusted HR (95% CI)^†^	P_trend_	Cases	Person-years	Multivariate-adjusted HR (95% CI)^†^	P_trend_
Pack-years smoked in 1989 (years)								
0	67	529,315	1.00 (ref)	0.88	310	528,862	1.00 (ref)	0.009
1–4.9	10	57,758	1.45 (0.74–2.83)		35	57,692	1.06 (0.75–1.51)	
5–14.9	14	129,899	0.83 (0.46–1.49)		97	129,822	1.24 (0.99–1.56)	
≥ 15	11	79,262	1.29 (0.67–2.50)		80	78,981	1.72 (1.34–2.22)	
Caffeine Intake (mg/day), 1991^@^								
< 100	27	217,522	1.00 (ref)	0.30	158	217,311	1.00 (ref)	0.02
100–249.9	27	192,943	1.10 (0.64–1.89)		147	192,694	1.02 (0.82–1.28)	
250–399.9	19	135,232	1.14 (0.62–2.10)		75	135,006	0.70 (0.53–0.93)	
≥ 400	8	114,914	0.54 (0.24–1.23)		81	114,485	0.82 (0.62–1.08)	
Alcohol Intake (g/day), 1991^@^								
Non-drinker	40	263,831	1.00 (ref)	0.38	204	263,266	1.00 (ref)	0.005
< 5	26	266,347	0.65 (0.40–1.08)		184	265,949	0.90 (0.73–1.10)	
5–14.9	10	104,562	0.62 (0.30–1.25)		69	104,517	0.85 (0.64–1.13)	
≥ 15	5	25,872	1.33 (0.51–3.50)		4	25,765	0.19 (0.07–0.52)	
Hormone therapy use in 1989								
Pre-menopausal	83	652,571	1.00 (ref)		362	652,448	1.00 (ref)	
Never user	4	39,869	0.91 (0.31–2.72)**	0.86***	42	39,615	1.01 (0.70–1.46)**	0.96***
Past/current use	14	101,764	1.42 (0.73–2.77)**	0.31***	115	101,295	1.47 (1.12–1.92)**	.005***
Menopausal status in 1989								
Pre-menopausal	77	599,632	1.00 (ref)		304	599,716	1.00 (Ref)	
Postmenopausal	19	143,662	2.05 (0.22–18.93)	0.53***	160	142,908	1.74 (0.55–5.53)	0.35***
Status unknown/unsure	0	N/A	N/A	0.36***	39	35,812	1.43 (0.99–2.06)	0.06***
Oral contraceptive use duration in 1989 (HR for every 1 year of use)			1.001 (0.999–1.002)				1.00 (0.99–1.00)	0.63***

HR, hazard ratio.

^†^Adjusted simultaneously for age, physical activity (quartiles), body mass index (BMI) (< 18.5, 18.5–24.9, 25–29.9, ≥ 30), and smoking status (never, past, current), with the exception of analyses of physical activity [metabolic equivalents (METs)], BMI and smoking which are not adjusted for the exposure in question.

*The total number of cases of CDC characterized ME/CFS was 102, but because of missing values, the numbers for some variables do not add up to 102.

**Hormone therapy use and menopausal status additionally adjusted for each other. Hormone therapy models adjusted for menopausal status and menopausal status models adjusted for hormone therapy use.

^@^Number of cases is smaller for caffeine and alcohol intake than for other variables, because caffeine and alcohol data was collected in 1991.

***Because of the categorical nature of the variables, we give the Wald p-value provided by the Cox model indicating whether the HR comparing the category in question is significantly different from 1, relative to the reference.

#### Physical activity

In general, physical activity—both at ages 18–22 and in adulthood—was not associated with risk for either ME/CFS or Severe Fatigue. The amount of time spent sitting at home was marginally associated with elevated risk of ME/CFS and of Severe Fatigue (Table 3).

#### Smoking

Current and past smoking was significantly associated with Severe Fatigue, but was not associated with the risk of ME/CFS (Table 3).

#### Caffeine

High intake of caffeine was associated with reduced risk of both ME/CFS and Severe Fatigue, although the association was significant only for Severe Fatigue (Table 3).

#### Alcohol

Intake of alcohol was unrelated to the risk of ME/CFS, and inversely related to the risk of Severe Fatigue (Table 3).

#### Hormone therapy and menopausal status

Each was positively associated with risk of Severe Fatigue (Table 3). The association with hormone therapy persisted after adjustment for menopausal status, but neither hormone therapy nor menopausal status was related to the risk of ME/CFS.

#### Mononucleosis

As shown in Table 4, women who reported, in 2001, having ever been diagnosed with infectious mononucleosis had a non-significantly but substantially elevated risk of ME/CFS (HR 1.77) and a slightly elevated risk of Severe Fatigue (HR 1.28). The same was true of women who reported the onset of ME/CFS or Severe Fatigue at baseline, in 1989, or after 2001.

**Table 4:** Hazard ratios for ME/CFS and for severe fatigue after infectious mononucleosis.

	Hazard ratio (95% CI)^a^
ME/CFS	1.77 (0.87–3.61)
Severe fatigue	1.28 (0.98–1.66)

^a^Hazard ratios of ME/CFS (N = 40 cases) and of Severe Fatigue (N = 331 cases) developing in those with infectious mononucleosis compared to a referent group without infectious mononucleosis. Hazard ratios are adjusted for age, physical activity (quartiles), body mass index (BMI) (< 18.5, 18.5–24.9, 25–29.9, ≥ 30), and smoking status (never, past, current).

## Discussion

### Different risk factors for ME/CFS vs. severe fatigue

In surveys of the general population, or of people seeking medical care, the number of people who describe a state of severe chronic fatigue is many times larger than the number of people who meet criteria for ME/CFS^21–24^. This raises the question of whether ME/CFS is simply one end of the spectrum of people with severe chronic fatigue, or a qualitatively different condition.

In this study, we identified a group of individuals with ME/CFS and a five-fold larger group with Severe Fatigue from a population of over 40,000 women enrolled in a rigorous prospective observational health study, Nurses’ Health Study II. With the statistical power afforded by a large sample, we then examined whether factors that increased the risk of membership in each of the two groups were similar—which might suggest that ME/CFS is a qualitatively similar but quantitatively more severe form of Severe Fatigue. Instead, we found that several risk factors for the two groups were quite different, suggesting that ME/CFS is different condition from Severe Fatigue.

### Risk factors distinguishing severe fatigue from ME/CFS

#### Age

Although the ages of the groups were similar (Table 1), increasing age was strongly related to increased risk in the Severe Fatigue group whereas it was unrelated to risk in the ME/CFS group. Similar to our results, a Swedish twin registry found that age did not affect the risk of ME/CFS among women^25^.

#### Obesity

Although the BMI of the groups was similar, both at age 18 and in adulthood (Table 1), we found a strong relationship of BMI ≥ 25 to Severe Fatigue but no relationship to ME/CFS (Table 3).

In contrast to our findings, several previous studies have found an association between increased BMI and ME/CFS. A cross-sectional study of over 9000 people^26^; a study of 1685 adolescents^27^; a small retrospective study of children and adolescents^28^; and a cross-sectional, population-based, case–control study of 5630 people^29^ all found higher levels of either increased BMI or metabolic syndrome (of which obesity is one criterion) in people with ME/CFS. In comparing the results of our studies to the results of these studies, we note that our study size was larger, and that differing case definitions for ME/CFS were used for these studies.

In contrast to both our findings and the findings of the studies just above, one study compared 247 people with ME/CFS to previously collected data from the general population and found that people with ME/CFS were *less* likely to be obese^16^. Also, a study of 59,101 survey respondents^30^ and a longitudinal population-based survey involving 1880 adolescents found no association of obesity with persistent fatigue (there were few subjects with ME/CFS)^15^. However, it is unclear whether people with “persistent” fatigue in this study had fatigue as severe as those in our Severe Fatigue group. Finally, a longitudinal study of over 16,000 newborns followed for up to 30 years found no association between obesity at age 10 years and self-reported ME/CFS^13^.

Metabolomic studies over the past decade have found aberrant metabolism, particularly impairment in generating adenosine triphosphate (ATP) from oxygen, glucose, fatty acids and amino acids, as well as a general hypometabolic state, as summarized in two recent reviews^31,32^. These abnormalities could be connected bidirectionally to obesity.

#### Smoking

We found a strong relationship of smoking to Severe Fatigue, but no association with ME/CFS (Table 3). Previous studies also have had conflicting results: one a large population-wide study found that current or former smokers were more likely to self-report the diagnosis of ME/CFS^30^, another found no association of ME/CFS with smoking^14^, and a third found that significantly *fewer* males with ME/CFS smoked^16^.

#### Caffeine

We found that a modest intake of caffeine was associated with a decreased risk of Severe Fatigue, but that caffeine intake was not associated with ME/CFS (Table 3). Our failure to find an association between caffeine intake and ME/CFS has also been reported by others^14^. It is not surprising that people with Severe Fatigue sought the stimulant effect of caffeine^33,34^, and interesting that this was not true for those with ME/CFS. Perhaps caffeine is not an effective stimulant or produces unpleasant symptoms (such as irritability) in many people with ME/CFS.

#### Intake of alcoholic beverages

We found that people with greatly increased alcoholic intake (≥ 15 g/day) were at greater risk for Severe Fatigue, but not at greater risk for ME/CFS (Table 3). Our findings for ME/CFS accord with those of two large population-wide surveys in which the self-reported diagnosis of ME/CFS was more likely in those who drank little or no alcohol^26,30^, a finding confirmed by two other studies^14,16^. These results for alcohol may reflect reverse causation since many people with ME/CFS report an intolerance to alcohol. No studies have linked alcohol abuse to ME/CFS.

### Risk factors not distinguishing severe fatigue from ME/CFS

#### Physical activity

At ages 18–22, people who subsequently developed ME/CFS had been more likely to engage in strenuous physical activity for 7 or more months per year than did people in the other two groups (Table 1). However, people who subsequently developed ME/CFS also were more often sedentary (Table 1). Thus, we did not find that consistent physical activity at ages 18–22 was associated with either Severe Fatigue or ME/CFS (Table 3).

We assessed physical activity in later adulthood (after the onset of the illness, in people with ME/CFS) both by estimating METs/week and by hours/week spent sitting at home. By the former measure, physical activity was not significantly associated with either Severe Fatigue or ME/CFS. By the latter measure, there was a significant trend in the people with ME/CFS, an association more likely explained by the disease causing the inactivity rather than the inactivity causing the disease. Other studies have reported similar results^13,14,26,30,35^.

#### Use of postmenopausal hormones

We found that use of hormone therapy (adjusted for menopausal status) was associated with Severe Fatigue but was not associated with ME/CFS.

### Mononucleosis as a risk factor for ME/CFS and severe fatigue

As seen in Table 4, women who reported having been diagnosed with infectious mononucleosis had an elevated risk of both ME/CFS (HR 1.77 [0.87–3.61]) and Severe Fatigue (HR 1.28 [0.98–1.66]). Given the small number of subjects with both a history of infectious mononucleosis and ME/CFS and Severe Fatigue, the power was poor to recognize the increased risk as significant.

#### Severe fatigue following mononucleosis

Multiple studies have found that a state of chronic fatigue sufficiently severe to impair functional status may linger for many months following mononucleosis^6,36–41^. Several risk factors for a state of chronic fatigue following mononucleosis, detectable during the acute illness, include: (1) symptoms consistent with dysautonomia including sensory sensitivity and increased pain^39,40^; (2) biomarkers of low-grade chronic inflammation^39,41^; and (3) T-cell activation^41^.

#### ME/CFS following mononucleosis

A chronic, fatiguing illness that fully meets criteria for ME/CFS also has been reported following mononucleosis, occurring in 7–23% of patients followed systematically for at least six months^5–12^. Chronic fatiguing illness occurs much more often following mononucleosis than after other infectious illnesses^5,42,43^. A past history of more frequent infectious diseases is reportedly a risk factor for ME/CFS following mononucleosis^4^. Certain symptoms during acute mononucleosis also appear to be risk factors, including greater severity of fatigue^8,44^, and gastrointestinal and other symptoms consistent with dysautonomia^10,12,45,46^. Because these symptoms suggesting dysautonomia are present during the first weeks of illness in previously healthy teenagers and young adults (they do not first appear only after months of inactivity), the dysautonomia is not explained by inactivity^45^. Autonomic dysfunction also often is present in ME/CFS that is not associated with mononucleosis, particularly among adolescents^47,48^.

#### Pathophysiology of fatigue states following mononucleosis

Several immunologic abnormalities during acute illness appear to be risk factors for subsequent ME/CFS: higher levels of interleukin-12 (a proinflammatory cytokine) and lower levels of interleukin-5 and interleukin-13 (anti-inflammatory cytokines)^12,46^; a particular pattern of cytokines^9^; cytokine networks that are disconnected from interferon-gamma signaling, possibly indicating a vulnerability to viral infections^49^; and deficient EBV-specific B- and T-cell memory^50^.

Finally, lower levels of adrenocorticotropic hormone (ACTH) 6 months following mononucleosis are found in people who develop ME/CFS than in those who recover^11^.

In contrast, people with ME/CFS following mononucleosis are no more likely to have higher measures of stress, anxiety or depression, when compared to those who fully recovered from mononucleosis^12^.

In summary, our study is only the latest to link mononucleosis and ME/CFS. What is different about our study is that previous studies typically have followed patients with mononucleosis for only a few years; moreover, many have shown that rates of ME/CFS decline with time^9,11^. For that reason, we followed patients for 20 years, and found that the link between mononucleosis and ME/CFS persists for many years, since the study participants who reported ME/CFS were (on average) in their fifties whereas mononucleosis typically occurs during adolescence or young adulthood.

By far the most common trigger of mononucleosis is acute primary infection with Epstein-Barr virus (EBV). In the past, a syndrome much like what we today call ME/CFS has been linked to serological evidence of reactivated infection with EBV in both sporadic^51–53^ and epidemic form^54^. One mechanism by which EBV infection might lead to ME/CFS involves an EBV protein, deoxyuridine triphosphate nucleotidohydrolase (dUTPase). This protein induces neuroinflammation and lethargy in female mice. Moreover, some people with ME/CFS have significantly elevated antibodies against dUTPase, the levels of which correlate with the severity of symptoms, and which distinguish people with ME/CFS as a group from healthy control subjects^55–57^.

Finally, a recent study has strongly suggested that EBV infection is a leading cause of multiple sclerosis (MS)^58^. Debilitating, chronic fatigue is a cardinal symptom of MS as well as ME/CFS. Moreover, people with ME/CFS often have areas of high signal in the white matter on T2-weighted magnetic resonance images (MRI), as do people with MS, although the areas are punctate rather than plaque-like and are located in subcortical rather than peri-ventricular areas^59^.

### Limitations

The study cohort was restricted to primarily white, educated, middle or upper-middle-class professional women, which may influence the generalizability of our results: some studies have shown a higher prevalence of ME/CFS among non-whites^22,60,61^.

We were able to assess risk factors both before and after the onset of ME/CFS. Exposure misclassification is possible, as all study exposures also were assessed by self-report on the NHS II questionnaire. However, study participants reported on these exposures repeatedly from the outset of the study cohort in 1989, well before reporting on symptoms of ME/CFS or Severe Fatigue in 2009. Any such exposure misclassification would be expected to be non-differential, potentially biasing estimates towards the null.

In contrast, an important limitation of this study is the retrospective ascertainment of the outcomes—time to ME/CFS and Severe Fatigue—which was based on a one-time questionnaire administered in 2009. The questionnaire asked participants to recall fatigue onset many years in the past, potentially leading to misclassification of the outcome. Furthermore, some cases of ME/CFS fatigue may have resolved by the time the questionnaire was administered and thus would not have been reported.

## Conclusions

In this large prospective cohort of female nurses who were followed for twenty years, we found that ME/CFS and Severe Fatigue had different risk factors, suggesting that the biological roots of ME/CFS may be different from those of the much more common state of Severe Fatigue. As have previous investigators, we also found an increased risk of ME/CFS in people with a history of infectious mononucleosis.

## Data Availability

The study protocol and statistical code supporting the conclusions of this article will be made available, without undue reservation, by Dr. Palacios (e-mail, palacios@hsph.harvard.edu).

## Abbreviations

ME/CFS: Myalgic encephalomyelitis/chronic fatigue syndrome
US: United States
HR: Hazard ratio
CI: Confidence interval
BMI: Body mass index
CDC: Centers for Disease Control and Prevention
NHS II: Nurses’ Health Study II
IRB: Institutional Review Board
ACTH: Adrenocorticotropic hormone
EBV: Epstein-Barr virus
dUTPase: Deoxyuridine triphosphate nucleotidohydrolase
MS: Multiple sclerosis
METs: Metabolic equivalents

## References

[1] Institute of Medicine. *Beyond Myalgic Encephalomyelitis/Chronic Fatigue Syndrome: Redefining an Illness.* (The National Academies Press, 2015). 25695122

[2] Reynolds KJ, Vernon SD, Bouchery E, Reeves WC (2004). The economic impact of chronic fatigue syndrome. Cost Effect Resour. Allocation.

[3] Komaroff AL (2019). Advances in understanding the pathophysiology of chronic fatigue syndrome. JAMA.

[4] Lacerda EM, Geraghty K, Kingdon CC, Palla L, Nacul L (2019). A logistic regression analysis of risk factors in ME/CFS pathogenesis. BMC Neurol..

[5] White PD (1998). Incidence, risk and prognosis of acute and chronic fatigue syndromes and psychiatric disorders after glandular fever. Br. J. Psychiatry.

[6] Buchwald DS, Rea TD, Katon WJ, Russo JE, Ashley RL (2000). Acute infectious mononucleosis: Characteristics of patients who report failure to recover. Am. J. Med..

[7] Hickie I (2006). Post-infective and chronic fatigue syndromes precipitated by viral and non-viral pathogens: Prospective cohort study. BMJ.

[8] Katz BZ, Shiraishi Y, Mears CJ, Binns HJ, Taylor R (2009). Chronic fatigue syndrome after infectious mononucleosis in adolescents. Pediatrics.

[9] Broderick G (2012). Cytokine expression profiles of immune imbalance in post-mononucleosis chronic fatigue. J. Transl. Med..

[10] Jason LA (2014). Predictors of post-infectious chronic fatigue syndrome in adolescents. Health Psychol. Behav. Med..

[11] Harvey JM (2016). Tracking post-infectious fatigue in clinic using routine Lab tests. BMC Pediatr..

[12] Jason LA, Cotler J, Islam MF, Sunnquist M, Katz BZ (2021). Risks for developing myalgic encephalomyelitis/chronic fatigue syndrome in college students following infectious mononucleosis: A prospective cohort study. Clin. Infect. Dis..

[13] Viner R, Hotopf M (2004). Childhood predictors of self reported chronic fatigue syndrome/myalgic encephalomyelitis in adults: National birth cohort study. BMJ.

[14] Hamaguchi M, Kawahito Y, Takeda N, Kato T, Kojima T (2011). Characteristics of chronic fatigue syndrome in a Japanese community population: Chronic fatigue syndrome in Japan. Clin. Rheumatol..

[15] Viner RM (2008). Longitudinal risk factors for persistent fatigue in adolescents. Arch. Pediatr. Adolesc. Med..

[16] Goedendorp MM, Knoop H, Schippers GM, Bleijenberg G (2009). The lifestyle of patients with chronic fatigue syndrome and the effect on fatigue and functional impairments. J. Hum. Nutr. Diet.

[17] Hernan MA, Olek MJ, Ascherio A (1999). Geographic variation of MS incidence in two prospective studies of US women. Neurology.

[18] Palacios N (2017). Exposure to particulate matter air pollution and risk of multiple sclerosis in two large cohorts of US nurses. Environ. Int..

[19] Palacios N, Fitzgerald KC, Komaroff AL, Ascherio A (2017). Incidence of myalgic encephalomyelitis/chronic fatigue syndrome in a large prospective cohort of U.S. nurses. Fatigue Biomed. Health Behav..

[20] Fukuda K (1994). The chronic fatigue syndrome: A comprehensive approach to its definition and study. Ann. Intern. Med..

[21] Buchwald D (1995). Chronic fatigue and the chronic fatigue syndrome: Prevalence in a Pacific Northwest health care system. Ann. Intern. Med..

[22] Reyes M (2003). Prevalence and incidence of chronic fatigue syndrome in Wichita, Kansaa. Arch. Intern. Med..

[23] Jason LA (1998). Estimating the prevalence of chronic fatigue syndrome among nurses. Am. J. Med..

[24] Jason LA (1999). A community-based study of chronic fatigue syndrome. Arch. Intern. Med..

[25] Evengård B, Jacks A, Pedersen NL, Sullivan PF (2005). The epidemiology of chronic fatigue in the Swedish twin registry. Psychol. Med..

[26] van't-Leven M, Zielhuis GA, van der-Meer JW, Verbeek AL, Bleijenberg G (2010). Fatigue and chronic fatigue syndrome-like complaints in the general population. Eur. J. Public Health.

[27] Norris T, Hawton K, Hamilton-Shield J, Crawley E (2017). Obesity in adolescents with chronic fatigue syndrome: An observational study. Arch. Dis. Child..

[28] Petrov D, Marchalik D, Sosin M, Bal A (2012). Factors affecting duration of chronic fatigue syndrome in pediatric patients. Indian J. Pediatr..

[29] Maloney EM, Boneva RS, Lin JM, Reeves WC (2010). Chronic fatigue syndrome is associated with metabolic syndrome: Results from a case-control study in Georgia. Metabolism.

[30] Rusu C, Gee ME, Lagace C, Parlor M (2015). Chronic fatigue syndrome and fibromyalgia in Canada: Prevalence and associations with six health status indicators. Health Promot. Chronic Dis. Prev. Can..

[31] Paul BD, Lemle MD, Komaroff AL, Snyder SH (2021). Redox imbalance links COVID-19 and myalgic encephalomyelitis/chronic fatigue syndrome. Proc. Natl. Acad. Sci. USA.

[32] Komaroff AL, Lipkin WI (2021). Insights from myalgic encephalomyelitis/chronic fatigue syndrome may help unravel the pathogenesis of postacute COVID-19 syndrome. Trends Mol. Med..

[33] Ferre S (2008). An update on the mechanisms of the psychostimulant effects of caffeine. J. Neurochem..

[34] Adan A, Prat G, Fabbri M, Sanchez-Turet M (2008). Early effects of caffeinated and decaffeinated coffee on subjective state and gender differences. Prog. Neuropsychopharmacol. Biol. Psychiatry.

[35] Newton JL (2011). Physical activity intensity but not sedentary activity is reduced in chronic fatigue syndrome and is associated with autonomic regulation. QJM.

[36] Smith MS (1991). Chronic fatigue in adolescents. Pediatrics.

[37] Feder HM, Dworkin PH, Orkin C (1994). Outcome of 48 pediatric patients with chronic fatigue: A clinical experience. Arch. Fam. Med..

[38] Rea TD, Russo JE, Katon W, Ashley RL, Buchwald DS (2000). Prospective study of the natural history of infectious mononucleosis caused by Epstein-Barr virus. J. Am. Board Fam. Pract..

[39] Pedersen M (2019). Predictors of chronic fatigue in adolescents six months after acute Epstein-Barr virus infection: A prospective cohort study. Brain Behav. Immun..

[40] Pedersen M (2019). Fatigue in Epstein-Barr virus infected adolescents and healthy controls: A prospective multifactorial association study. J. Psychosom. Res..

[41] Fevang B (2021). Lasting immunological imprint of primary Epstein-Barr virus infection with associations to chronic low-grade inflammation and fatigue. Front. Immunol..

[42] Wessely S (1995). Postinfectious fatigue: Prospective cohort study in primary care. Lancet.

[43] Bakken IJ (2016). Comorbidities treated in primary care in children with chronic fatigue syndrome/myalgic encephalomyelitis: A nationwide registry linkage study from Norway. BMC Fam. Pract..

[44] Katz BZ (2019). A validated scale for assessing the severity of acute infectious mononucleosis. J. Pediatr..

[45] Katz BZ, Stewart JM, Shiraishi Y, Mears CJ, Taylor R (2011). Autonomic symptoms at baseline and following infectious mononucleosis in a prospective cohort of adolescents. Arch. Pediatr. Adolesc. Med..

[46] Jason LA, Cotler J, Islam MF, Furst J, Katz BZ (2022). Predictors for developing severe myalgic encephalomyelitis/chronic fatigue syndrome following infectious mononucleosis. J. Rehab. Therapy.

[47] Bou-Holaigah I, Rowe PC, Kan J, Calkins H (1995). The relationship between neurally mediated hypotension and the chronic fatigue syndrome. JAMA.

[48] Rowe PC (2002). Orthostatic intolerance and chronic fatigue syndrome: New light on an old problem. J. Pediatrics.

[49] Jason LA (2021). Cytokine networks analysis uncovers further differences between those who develop myalgic encephalomyelitis/chronic fatigue syndrome following infectious mononucleosis. Fatigue Biomed. Health Behav..

[50] Loebel M (2014). Deficient EBV-specific B- and T-cell response in patients with chronic fatigue syndrome. PLoS ONE.

[51] Jones JF (1985). Evidence for active Epstein-Barr virus infection in patients with persistent, unexplained illnesses: Elevated anti-early antigen antibodies. Ann. Intern. Med..

[52] Straus SE (1985). Persisting illness and fatigue in adults with evidence of Epstein-Barr virus infection. Ann. Intern. Med..

[53] DuBois RE (1984). Chronic mononucleosis syndrome. South Med. J..

[54] Buchwald D (1992). A chronic illness characterized by fatigue, neurologic and immunologic disorders, and active human herpesvirus type 6 infection. Ann. Intern. Med..

[55] Williams MV, Cox B, Lafuse WP, Ariza ME (2019). Epstein-Barr virus dUTPase induces neuroinflammatory mediators: Implications for myalgic encephalomyelitis/chronic fatigue syndrome. Clin. Ther..

[56] Halpin P (2017). Myalgic encephalomyelitis/chronic fatigue syndrome and Gulf War illness patients exhibit increased humoral responses to the herpesviruses-encoded dUTPase: Implications in disease pathophysiology. J. Med. Virol..

[57] Lerner AM (2012). Antibody to Epstein-Barr virus deoxyuridine triphosphate nucleotidohydrolase and deoxyribonucleotide polymerase in a chronic fatigue syndrome subset. PLoS ONE.

[58] Bjornevik K (2022). Longitudinal analysis reveals high prevalence of Epstein-Barr virus associated with multiple sclerosis. Science.

[59] Schwartz RB (1994). Detection of intracranial abnormalities in patients with chronic fatigue syndrome: Comparison of MR imaging and SPECT. AJR Am. J. Roentgenol..

[60] Steele L (1998). The epidemiology of chronic fatigue in San Francisco. Am. J. Med..

[61] Dinos S (2009). A systematic review of chronic fatigue, its syndromes and ethnicity: Prevalence, severity, co-morbidity and coping. Int. J. Epidemiol..

